# Community-based organization perspectives on participating in state-wide community canvassing program aimed to reduce COVID-19 vaccine disparities in California

**DOI:** 10.1186/s12889-023-16210-9

**Published:** 2023-07-14

**Authors:** Lisa N. Mansfield, Savanna L. Carson, Nisha Sunku, Alana Troutt, Shelli Jackson, David Santillan, Stefanie D. Vassar, Dale Slaughter, Gloria Kim, Keith C. Norris, Arleen F. Brown

**Affiliations:** 1 Division of General Internal Medicine and Health Services Research, Department of Medicine, David Geffen School of Medicine, University of California, Los Angeles, CA USA; 2 Present Address: School of Nursing, University of North Carolina at Chapel Hill, Chapel Hill, NC 27599 USA; 3 Olive View-UCLA Medical Center, Sylmar, CA USA; 4 California Government Operations Agency, State of California, Sacramento, CA USA; 5 Present Address: San Francisco Health Plan, San Francisco, California United States; 6 Blue Phoenix Strategies, Falls Church, VA USA

**Keywords:** COVID-19, COVID-19 vaccination, Health disparities, Health equity, Community-engaged research, Race/ethnicity, Public health, Community health

## Abstract

**Background:**

Inequities in COVID-19 vaccine accessibility and reliable COVID-related information disproportionately affected marginalized racial and ethnic communities in the U.S. The Get Out the Vaccine (GOTVax) program, an innovative statewide government-funded COVID-19 vaccine canvassing program in California, aimed to reduce structural barriers to COVID-19 vaccination in high-risk communities with low vaccination rates. GOTVax consisted of a community-academic-government partnership with 34 local trusted community-based organizations’ (CBOs) to conduct COVID-19 vaccine outreach, education, and vaccine registration. The purpose of this qualitative evaluation study was to explore the barriers and facilitators of using local CBOs to deploy a geographically, racially, and ethnically diverse state-wide COVID-19 vaccine outreach program.

**Methods:**

Semi-structured online interviews were conducted with participating GOTVax CBO leaders from November 2021 to January 2022. Transcripts were analyzed using reflexive thematic analysis.

**Results:**

Thirty-one of 34 CBOs participated (91% response rate). Identified themes encompassed both facilitators and barriers to program participation. Key facilitators included leveraging trust through recognized entities; promoting empathetic, tailored outreach; and flexibility of milestone-based CBO funding contracts for rapid program implementation. Barriers included navigating community sociopolitical, geographic, and cultural factors; managing canvassers’ safety; desiring metrics for self-evaluation of outreach success; mitigating canvassing technology challenges; and concerns of program infrastructure initially limiting outreach. CBOs problem-solved barriers with academic and government partners.

**Conclusions:**

Between May and December 2021, the GOTVax program reached over 2 million California residents and registered over 60,000 residents for COVID-19 vaccination. Public health campaigns may improve benefits from leveraging the expertise of community-trusted CBOs and universities by providing flexible infrastructure and funding, allowing CBOs to seamlessly tailor outreach most applicable to local minoritized communities.

**Supplementary Information:**

The online version contains supplementary material available at 10.1186/s12889-023-16210-9.

## Introduction

SARS-CoV-2 (COVID-19) infection, hospitalization, and death rates have disproportionately affected marginalized racial and ethnic (minoritized) populations across the U.S [1]. In Los Angeles County, the most populous and diverse county in California, minoritized groups such as Hispanic/Latinx, Black/African American, Pacific Islander, and Native American have the highest rates of COVID-19 cases, hospitalizations, and deaths [2]. COVID-19 disparities are worse among residents living in high-poverty, rural areas and individuals lacking healthcare insurance or delay seeking care [2]. Despite the promise of vaccines and therapeutics, early vaccine disparities were noted and initially attributed to historical medical and government mistrust in racial and ethnic minoritized communities [3, 4]. However, subsequent evidence implicated other factors in COVID-19 vaccine disparities: structural barriers to accessing vaccines; variable access to reliable, accurate, linguistically- and culturally-relevant COVID-related information [3–11]; contemporary medical and government mistrust; and vaccine scheduling strategies that were heavily reliant on technology in communities with limited health literacy and internet access [3, 5, 10, 12–14].

Community-engaged strategies are widely proposed for COVID-19 vaccine outreach [15, 16], prioritizing community-academic partnerships and mobilizing trusted community-based organizations (CBOs). Community-engaged outreach may enhance our understanding of how to reduce structural, communication, and technology barriers to COVID-19 vaccination from individuals with lived experiences and knowledge of the community’s needs, preferences, norms, and resources [4, 6, 11, 15–19]. However, few studies have explored CBOs’ perspectives on how government and academic program design, funding, training, and other factors may impact their ability to conduct community-engaged outreach. We describe a qualitative evaluation of the barriers and facilitators of using local CBOs during a pandemic to deploy a geographically, racially, and ethnically diverse state-wide COVID-19 vaccine outreach program in California. The Get Out the Vaccine (GOTVax) program arose from California NIH Community Engagement Alliance. GOTVax aimed to reduce structural barriers to COVID-19 vaccination by contracting with local CBOs to conduct on-the-ground canvassing for COVID-19 outreach, education, and vaccine registration in high-risk communities.

## Methods

### Program design and structure

The GOTVax program was a novel community-academic-government partnership between the State of California’s Office of Government Operations (funder), the University of California, Los Angeles (UCLA: funding and contract administrators and COVID-19 vaccine education training facilitators), and 34 local CBOs across California (canvassing managers). GOTVax leveraged California’s Get Out The Vote (GOTVote) voter outreach efforts using door-to-door canvassing, phone banking, and text messaging. This GOTVote strategy allowed the GOTVax program to utilize existing infrastructure rapidly, including canvassing tracking technology (Political Data Intelligence [PDI] software and the California state-wide vaccine registration “MyTurn” website). Between May and December 2021, the GOTVax CBOs helped to reach over 2 million residents and registered over 60,000 for COVID-19 vaccination.

**Fig. 1:**
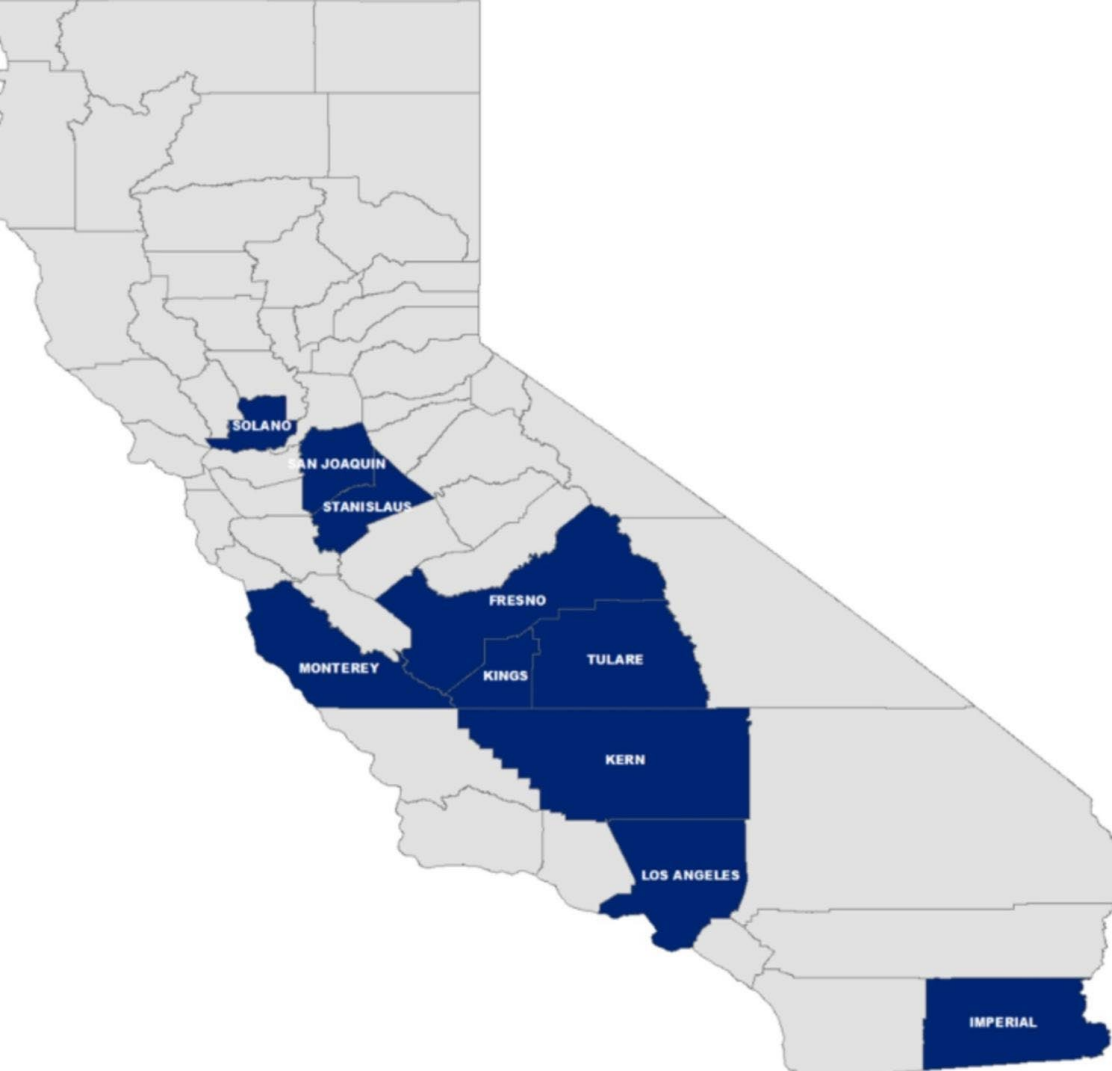
Selected Counties for GOTVax Program in California

State-designated GOTVax canvassing areas included Los Angeles County and eight counties in the Central Valley in California (Fig. 1). These areas were high-risk zip codes (a) disproportionately impacted by COVID-19, (b) in the lowest quartile of the California Healthy Places Index, a database that depicts community conditions that predict life expectancy and influence health [20] and (c) unreached by other state programs. The government and academic partners identified CBOs that provided services to communities in the selected high-risk zip codes and invited them for program participation. The State of California’s Office of Government Operations launched a new milestone-based funding mechanism that provided CBOs initial ramp-up funding and then disbursed funds based on their program performance. See **Appendix A** for additional GOTVax program information.

Local CBOs contracted with GOTVax to conduct regional canvassing, facilitating COVID-19 vaccination sign-up, education, and pandemic resources. CBOs were encouraged to hire canvassers and managers who were unemployed or underemployed residents from the selected communities. Canvassers and canvassing managers participated in training sessions led by government and academic partners on COVID-19, how to share accurate information about COVID-19 vaccination in multi-languages, canvasser safety, commonly asked questions and PDI technology.

Outreach strategies and materials were designed, developed, and implemented in collaboration with the CBOs and informed by focus groups with multiethnic communities in Los Angeles County on structural barriers to COVID-19 prevention and vaccine information, accessibility, and registration [21]. Factors influencing COVID-19 vaccine accessibility and acceptability from the focus groups were used to inform: (a) the training for CBO leaders and canvassers, (b) the process and strategies developed by the CBOs, UCLA, and the State of California to reduce structural barriers to vaccine hesitancy, and vaccine accessibility, and (c) the development of effective vaccine messaging and resources to combat misinformation and address the community’s vaccine concerns.

### Study methods

This study used a qualitative descriptive design using online, semi-structured interviews with leaders in participating GOTVax CBOs to understand their perspectives on managing and implementing activities for COVID-19 vaccine outreach. IRB approval was obtained from the UCLA Institutional Review Board.

### Recruitment

CBOs were asked to identify leaders within their organization for interview participation. Eligibility criteria included (a) employment as a CBO GOTVax leader (i.e., an executive director or manager responsible for managing and implementing the GOTVax activities), (b) spoke English or Spanish, and (c) able to speak by phone/Zoom in an online group setting for a 45 to 60-minute time interval. One to three GOTVax leaders were recruited per CBO to participate in a combined interview.

Recruitment occurred via telephone and email. A recruitment letter, study information sheet, and flyer were emailed to all CBOs to disseminate to their executive directors and GOTVax manager. Participants contacted the study team by responding to the recruitment flyer via email or telephone and were screened for eligibility. Eligible participants scheduled an online interview and received a link to a pre-interview demographic survey.

### Interview guide

Semi-structured online interviews with CBO executive directors or managers were conducted via Zoom from November 2021 to January 2022. We invited all 34 contracted GOTVax CBOs. Participants were asked about services and operations their CBO provided before the COVID-19 pandemic, the impact of the pandemic on CBO operations and actions taken to respond to the community’s needs, helpful strategies in implementing and managing GOTVax operations, and challenges CBOs experienced in implementing and managing GOTVax activities and operations (see Table 1 for interview guide). Interviews were completed within 45–60 min and offered in English or Spanish. Verbal consent was obtained at the beginning of the interview. The most senior leader within each organization completed a pre-interview demographic survey. After the interview, each organization received one $250 Amazon gift card for their participation.

**Table 1:** Interview Guide with CBO Leaders

Questions
1. Tell me about your organization. What did your organization do before the COVID-19 pandemic?
2. Describe what motivated your organization to participate in the GOTVax program.
3. What helped the ramp-up and managing of the GOTVax activities at your organization?
4. What were some of the challenges in the ramp-up and managing the GOTVax activities at your organization?
5. What strategies has your organization found helpful to engage people when canvassing for COVID-19 vaccine outreach in your communities?
6. What does your organization need to address COVID-19 vaccine misinformation in the communities you serve?
7. Briefly describe in what ways was your organization impacted by the COVID-19 pandemic.
8. As your organization recovers from the pandemic, describe your organization’s greatest needs for the communities that you serve.
9. What ideas do you have to improve the GOTVax program?
10. Is there anything else you would like to add that we might not have asked you about or do you have any questions?

### Data analysis

All interviews were recorded, professionally transcribed, and analyzed using Atlas.Ti, version 9 software. Transcripts were checked for accuracy, de-identified, and systematically analyzed using qualitative methods. Interview transcriptions were coded using inductive six-phase reflexive thematic analysis [22, 23]. Two researchers with expertise in qualitative methods (LNM, SLC) worked collaboratively to analyze themes, develop thematic structure, and verify theme appropriateness. Themes and subthemes were mapped, revised, and refined to ensure a good fit with the data. The two researchers performed comparative analysis of consistencies, inconsistencies, and frequency between interviews to determine areas of significance. Coding decisions were tracked in coding memos. For the demographic CBO survey, descriptive statistics were conducted to describe the characteristics of the participating CBOs. One participating CBO consisted of two organizations that merged together for the GOTVax program with each completing a demographic survey, totaling 32 responses.

## Results

Among 34 invited CBOs, 31 CBOs participated in an interview (91% response rate), totaling 45 participants. Most CBOs were operating for more than 15 years (59.4%), employed less than 25 employees (56.3%), provided services to more than 10,000 people in their communities (53.1%), and had an annual budget of $1 million to $9 million (43.8%) (Table 2). Before involvement in the GOTVax program, most CBOs did not receive COVID-related financial support during the pandemic (55%) yet were involved in COVID-19-related outreach that promoted events about COVID-19 or provided COVID-19 and vaccine education (28.8%, respectively).

**Table 2:** CBO Characteristics, N = 31^a^

Characteristics		n (%)
Years of Operation		
1 to 5 years		5 (15.6%)
6 to 10 years		6 (18.8%)
11 to 15 years		2 (6.3%)
More than 15 years		19 (59.4%)
Number of Employees		
Fewer than 25		18 (56.3%)
25 to 50		6 (18.8%)
51 to 99		2 (6.3%)
100 to 200		1 (3.1%)
More than 200		5 (15.6%)
Number of People Services Provided to Yearly		
Less than 1,000		5 (15.6%)
1,001 to 5,000		7 (21.9%)
5,001 to 9,999		1 (3.1%)
10,000 or more		17 (53.1%)
Other (Please Specify):		1 (3.1%)
Unsure		1 (3.1%)
Annual Budget		
Less than 1 million		13 (40.6%)
$1 million to $9 million		14 (43.8%)
$10 million to $100 million		3 (9.4%)
Other		1 (3.1%)
Unsure		1 (3.1%)
Previous COVID-Related Outreach		
Yes		23 (71.9%)
No		8 (25.0%)
Unsure		1 (3.1%)
Type of Organization (Select all that apply)		
Faith-Based Organization		1 (2.6%)
Multipurpose Social Service Organization		17 (42.5%)
Housing		1 (2.6%)
University-Affiliated Community Service Organization		1 (2.6%)
Other (Please specify):		20 (50.0%)
Community-Based Non-Profit		14
Community Advocacy Organization		1
Community College		1
Environmental Equity Non-Profit Organization		1
Farmworkers and Low-Income family Assistance		1
Youth Program		2
Received financial support during the pandemic^b^		
Yes		10 (32.3%)
No		17 (54.8%)
Unsure		4 (12.9%)
Type of Previous COVID-Related Activities (Select all that apply)		
Designated as a COVID-19 vaccination site		4 (6.1%)
Helped with COVID-19 vaccine registration		14 (21.2%)
Promoted events about COVID-19		19 (28.8%)
Provided COVID-19 and vaccine education		19 (28.8%)
Served as a COVID-19 testing site		6 (9.1%)
Other (Please specify):		4 (6.1%)
Created website to support COVID education and making appointments, as well as testing		1
Provided PPE masks and hand sanitizers in outreach events		1
Hosted vaccine clinic for ag workers		1
Food and Mask distributions		1

*Note*: ^a^One participating CBO consisted of two organizations that merged together for the GOTVax program; leaders from each organization completed a demographic survey, totaling 32 sum responses. ^b^Financial support included receiving funding from any federal, state, or regional assistance programs during the pandemic, such as Paycheck Protection Program (PPP) loans, aids from the Center of Disease Prevention and Control, or the State of California

Identified themes reflecting facilitators to program participation included: leveraging trust through recognized entities, promoting empathetic, tailored outreach, and flexible milestone-based CBO contracts. Barriers included: navigating community sociopolitical, geographic, and cultural factors, managing canvassers’ safety, desiring performance metrics for self-evaluation, conducting rapid hiring of canvassers and training staff, mitigating canvassing technology challenges, and concerns of program infrastructure initially limiting outreach to registered voters (Table 3). Identified barriers were addressed during weekly check-ins with CBOs leaders and problem-solved with the academic and government partners. Strategies used to address some barriers are described within each theme.

**Table 3:** Facilitators and Barriers to Participation in the GOTVax Program among CBO Leaders

Facilitators	
Themes	Relevant Quotes
Leveraging trust through recognized entities	“*The trust that we had from the community already… Even when the vaccine came along, they trusted our opinion on whether you should or not get the vaccine*.” -CBO #26 “*It’s still a challenge because this is a community who has a lot of mistrust… especially with the government …because many of the community are undocumented immigrants… so the fact that we came to the community looking like them and speaking like them, presenting as ourselves from a non-profit organization and not from the office of the Governor or the Department of Public Health that made a big difference*.” -CBO #31
Promoting empathetic, tailored outreach	“*At the time, we had gotten a large amount of money for rental and utility bill assistance, so that was our approach… What we said was ‘We’re a nonprofit organization. We wanted to let you know about …services… then we hit them with that, ‘By the way, are you vaccinated? Are you interested?*’” -CBO #25 “*We created a survey ourselves to try to assess like what are folks [needing]. And what are they identifying as like barriers to accessing or addressing the COVID situation? …We understood the impact of COVID and so we tried to bring in resources that folks were soliciting*.” -CBO #13
Flexibility of milestone-based CBO contracts for rapid program implementation	“*What helped us with the UCLA grant was the requirements weren’t as stringent as working with the county, …federal government like HHS… that was a huge hurdle that we did not have to face. Everyone really ensured to make sure that our organization could do the work and get it done*.” -CBO #3 “*As a nonprofit, …[with] grants, the funding is always really delayed. So that can be tricky of estimating, when are you going to launch or… pay the employees… the good thing about this time was [UCLA was] able to disperse the money [initially] to the organizations*.” -CBO #17
**Barriers**	
Navigating community sociopolitical, geographic, and cultural factors	“*It was a struggle…a lot of people not willing or wanting to receive our messaging… And there was an ongoing campaign of anti-vaxxers in some of the areas*.” -CBO #8 “*It’s the middle of the summer, super-hot, for the Central Valley…the air quality was bad because there were forests fires… I think [UCLA] were very responsive in adjusting… so if it was too hot or the air quality was bad, then we can do phone banking*.” -CBO #28 “*The culture within those communities…L.A.’s so diverse every three blocks…so, we’re going into a different community that is way different where the people were coming from that there was an adapting, there was a different perspective.*” -CBO #22
Managing canvassers’ safety	“*We always allowed them to walk away if they didn’t feel safe. We also had one-on-one conversations with our canvassers to kind of identify who has gone to these areas before, who might feel, safe particularly in those areas. We tried to make sure we were matching, like, very young people with more older, established canvassers whenever possible so that it felt more safe*” -CBO #5 “*We, unfortunately live in a community where there’re different gangs… so what we did is we made and created our own shirts so that they stand out and folks know that these folks are working, they’re community members. We even went as far as getting them those safety vests, like the neon ones, just so they kind of stand out a little bit more*.” -CBO #11
Desiring performance metrics for self-evaluation of outreach success	“*I don’t know if the metrics were very clear. We kept asking, are we doing a good job? It was like how do we know? And to us, it was very important to make sure we were doing a good job…there was never any feedback on okay, you guys are below average on the response rates. What can we do to help? It was like, just report back to us and we’re good*.” -CBO #27
Conducting rapid hiring of canvassers and training staff	“*It was the fact that we didn’t have enough time to train them the way we train our staff. It was just like we have to start like right away*.” -CBO #9 “*For part-time workers, I think more than anything we want to take care of people, and we’re talking about health, then we should be providing health opportunities for people, like health insurances and the whole works*.” -CBO #20
Mitigating canvassing technology challenges	“*PDI is another issue. The way the application does not work on some of those phones, the way it gets stuck, the way it takes forever to load, the way it takes forever to update…all those also have to add to the problem… we’ve got to do manual entries on all this stuff*.” -CBO#23 “*The first round with the PDI, it was good because it was easy to report… and follow the tracker with the canvassers. But after that it was like five rounds, six rounds, seven rounds, nine rounds [of canvassing]…we just like, oh, there are no more houses [to knock on].”* -CBO#18
Concerns of program infrastructure initially limited outreach	“*At the beginning we were targeting the addresses that we had listed…[so] if we were to start with every single household…that would have kind of made it seem that we weren’t considering a particular part of the community over everybody*. -CBO#8 “*When we were using PDI…the promotoras [were] not agreeing with that way to knock on this door, and don’t know on the other door, because it’s not on the system for us was very frustrating. When we see, then, people who are not citizens, or don’t have a legal status have more difficulty to get any resource… next door, they probably are in more need.*” -CBO #10

### Facilitators

#### Leveraging trust through recognized entities

CBO leaders reported that leveraging their existing trusting relationship within their communities led to program successes, including making residents feel comfortable asking COVID-19 vaccine questions and expressing concerns. Hiring staff who were reflective of, and/or residents of, the canvassed communities also promoted trust. One leader described, “*The fact that we…look like them, speak like them. We come from the same countries. We are immigrants…that makes things a little bit easier*” (CBO #31).

Partnership with a well-recognized academic institution also helped leverage community trust to provide COVID-19 vaccine education. Leaders described how COVID-19 vaccine information and messaging training from the academic partner improved canvasser readiness to address the community’s vaccine concerns. One leader stated, “*Promotoras felt supported by having this literature that said UCLA on there… people listened… it’s a respected organization*” (CBO #28).

### Promoting empathetic, tailored outreach

CBO leaders highlighted canvasser storytelling to demonstrate understanding and empathy regarding the pandemic’s effects on the communities, which helped to ease COVID-19 vaccines conversations. By sharing how the pandemic affected their own families and communities and discussing their own personal vaccine experiences, the canvassers encouraged residents to voice their vaccine concerns in a non-judgmental manner while validating their vaccine deliberations. Compassionate conversations preceding sharing of factual, updated vaccine information were particularly influential in promoting informed decision-making. One leader shared, “*I [say] to them, ‘I understand your fears regarding the medical profession. I just want to make sure that you have all the information presented to you to make your proper decisions for yourself*’” (CBO #7).

CBO leaders described the importance of tailoring outreach to the community’s needs, including targeting local venues, promoting local vaccine pop-ups, and providing community resources beyond the household information provided in PDI. CBO leaders used canvassing outreach to assess for social needs and provide information and connections to needed services (e.g., rental relief and food insecurity). One CBO leader stated, “*We partnered [with] the city [to] launch the rental assistance piece…[assist with applications] for the guaranteed basic income program…[connecting people to] the nearest foodbank… additional resources other than [the] vaccine…*” (CBO #13).

### Flexibility of milestone-based cbo contracts for rapid program implementation

The rapid launch of GOTVax was facilitated by changes in standard funding protocols that allowed flexible funding, advanced disbursement of funds for outreach activities, and streamlined ramp-up. The State of California Office of Government Operations and UCLA created new and innovative funding mechanisms that disbursed initial funds at signing of contract and subsequent funds based on each CBOs’ performance and milestones achieved during the program instead of following a cost-reimbursement model. For most CBOs, the contracts provided leaders with guidance for quick ramp-up without strict requirements. A CBO leader stated, “*[Dispersing money to the organizations] allowed us to get the confidence to go out there, and we can pay everybody*” (CBO #17).

### Barriers

#### Navigating community sociopolitical, geographic, and cultural factors

CBO leaders felt that sociopolitical factors, geographic factors, and cultural incongruence in some communities impeded outreach. CBO leaders in rural locations described a tension between canvassers promoting and residents opposed to vaccination, which they attributed to politicizing the COVID-19 vaccine. One CBO leader stated, “*[There were things we faced] with the vaccinations and the resistance because it was made political…the canvassers faced [a lot of things] with racism, with anti-vaxxers*” (CBO #19).

Among CBOs located in urban communities, canvassers faced challenges that included limited access to residents in apartment buildings or gated residences, guard dogs, and gang violence. One CBO leader shared, “*We had incidents that involved guns and being threatened, an incident with M.S.13 or people just insulting you because you’re offering a vaccine appointment*” (CBO #31). CBOs in California’s Central Valley experienced challenges, needing to schedule canvassing in the early morning or evenings due to excessive heat. One CBO leader stated, “*Some days it was 116°. The iPads would turn off and say it’s too hot. We provided things like electrolytes… bandanas immersed in water so they can stay cool… change the time when they would be doing outreach outside, and then [converting to] phone banking”* (CBO #26). Remote locations of vaccine clinics and pharmacies and greater distances between houses also made it difficult to reach residents in rural communities. A CBO leader stated, “*The houses are two miles apart. Do you know how long it takes to walk two miles from one house to another?”* (CBO #23).

Canvassers in both urban and rural communities experienced harassment from residents and police interference due to residents perceiving canvassers as trespassing. One CBO leader shared, “*There were people [screaming] ‘Get off my lawn, I’m going to hose you down with the water… you’re not supposed to be here’*” (CBO #18). Another CBO leader shared, “*One [canvasser] actually did get arrested…for trespassing*” (CBO #9).

Although hiring canvassers with racial, ethnic, and cultural congruence with the communities served was a hallmark feature of the GOTVax program, some CBOs described challenges providing linguistically-congruent COVID-19 information and vaccine registration. One CBO leader stated, “*We had issues [in] certain areas where Mandarin is being spoken. How do we connect to be able to assist all these different needs?”* (CBO #22). Racial and ethnic differences among canvassers and residents sometimes led to discriminatory and racist encounters. A CBO leader described concerns when racial and ethnic divides occured, “*Most of the canvassers we hired were Latinos…but they were going to areas where there were primarily African Americans. So they were being asked, ‘What are you guys doing in our neighborhood?’*” (CBO #4).

### Managing canvassers’ safety

CBO leaders reported canvassing safety concerns and described common mitigation strategies. Several recommended ways to standardize canvasser clothing for easy professional identification when door-knocking, and CBOs proactively informed the local police about the government-funded outreach program. One CBO described, “*They had name badges and safety vests. We contacted our local police department and sheriff’s department… to let them know that we had canvassers out five days a week in case people did call the police on them* (CBO #5). Additionally, CBO leaders voiced canvassers safety concerns to the academic and government partners who worked with CBO leaders to problem-solve solutions to ensure canvasser safety during outreach. Some solutions included reassigning CBOs from dangerous areas to safer locations for outreach, adjusting canvassers’ schedules to conduct door-to-door outreach in the early evening to avoid excessive heat, and switching outreach to phone banking during high heat events.

### Desiring performance metrics for self-evaluation of outreach success

Although contracts were flexible and did not contain strict requirements for program participation, many CBO leaders expressed a desire for evaluation metrics to improve their GOTVax program performance. One CBO leader expressed ambiguity about their performance, wanting to know “on *a timely basis [whether] something is working for the organization or other strategies that we should consider to be more effective*” (CBO #13).

### Conducting rapid hiring of canvassers and training staff

CBO-required GOTVax staff included canvassers, field managers, and CBO managers. Although some CBOs could rehire community workers from previous outreach programs, the rapid ramp-up of the GOTVax program created challenges for many CBOs to hire and train staff quickly. Some CBOs did not have the administrative/human resources infrastructure to hire staff rapidly and required more time to launch outreach. One leader stated, “*It was the rush to hire people. I would rather have a little bit more time to well train, provide more resources, and capacity building to them*” (CBO #18). CBO leaders also raised concerns for retaining short-term staff, limited employee benefits and canvassers completing recommended weekly hours for a part-time job with competing life circumstance.

The academic-led training sessions and multi-language vaccine handouts were helpful to most CBO leaders preparing canvassers to address COVID-19 vaccine concerns. However, COVID-19 vaccine education quickly became outdated, with emerging COVID-19 variants and changing vaccine eligibility, resulting in some CBO leaders researching the latest COVID-19 vaccine information, retraining staff, and updating resources.

### Mitigating canvassing technology challenges

The PDI tracking technology used to measure the canvassers’ activity during outreach posed additional challenges for the CBOs. Many CBO leaders faced a steep technology learning curve, including needing an internet connection to access PDI during outreach for vaccine registration, daily system updates, and technology troubleshooting. One leader described, “*[In] rural [areas], you don’t have access to the internet…[which was needed for] the platform to be able to troubleshoot in real-time”* (CBO #19). Additionally, some leaders were dissatisfied with the boundaries of the communities they were assigned via PDI. A CBO leader described, “*There was not enough turf [houses in the assigned community] that was assigned to us, so we were doubling up and going, again, to those same locations”* (CBO #4).

### Infrastructure initially limited outreach

Households selected in the assigned zip codes for outreach were identified by the PDI app that included census, voter registration, and commercial data, which meant not all households in each zip code of focus were listed for outreach. CBOs evolved to use alternative tracking and outreach strategies in local areas to reach other groups. Many CBOs leaders expressed concern that initially relying solely on these data sources to identify eligible households created inequity in outreach and excluded populations, such as undocumented residents. One leader described, “*Using PDI was restrictive*…*We’re in a neighborhood, knock on every single house… let’s not restrict who we’re trying to reach out to*” (CBO #1). CBO leaders worked with the program’s government and academic partners to resolve this issue, expanding outreach activities to include crowd-canvassing and knocking on all doors in each community.

## Discussion

In this qualitative evaluation of the GOTVax program, CBOs reported how the novel GOTVax program helped them with rapid government funding, academic training, and program infrastructure to “*care for their community.*” Still, leaders also identified implementation challenges, CBO needs, and program gaps with implications for optimizing program design in future community-academic-government partnerships. CBO leaders identified several components of successful COVID-19 vaccine outreach, including leveraging community trust through recognized entities, promoting empathetic, tailored outreach, and flexible milestone-driven contracting to support implementation. The GOTVax CBOs also provided important insights into barriers to COVID-19 vaccine outreach, including navigating community sociopolitical and geographic factors, managing canvassers’ safety, requesting additional performance metrics to assess outreach success, rapidly hiring and training staff, mitigating canvassing technology challenges, and overcoming the initial program infrastructure restricting outreach to all households in the community.

The GOTVax program reinforced how community engagement may improve locally tailored, trusted outreach strategies for diverse communities [15, 16]. CBOs in this study recognized public health’s gap in active trust-building efforts [5, 24, 25], compounding existing health inequities [26]. Additionally, CBOs described challenges in tailoring outreach to some communities due to the heightened politicization of COVID-19 vaccines promoting public skepticism about government programs [27, 28]. Using trusted CBOs helped mitigate issues of trust between minoritized communities and public health and governmental entities [16, 29, 30]. Another community-academic-public health partnership utilized door-to-door canvassing tactics, established accessible, walk-up vaccination sites in local neighborhoods, and used Spanish media outlets to inform the community and reduce vaccine access barriers [16]. However, many of these studies show limited CBO insight in program evaluation. This study builds upon this prior work by providing unique insights into the perspectives of experienced CBO leaders whose agencies were mobilized to conduct vaccine outreach.

CBOs in this study described the importance of innovative, rapid and flexible financing mechanisms to reduce contracting barriers. These mechanisms were additionally facilitated by flexible government pandemic emergency funding parameters. To facilitate community partnerships, particularly in future public health emergencies, CBOs endorsed deliverable-based contracts with low requirements for initiating the awards (to avoid invoice-based/cost-reimbursement billing), flexible spending, including allotments for ramp-up/down activities, and support for capacity building, which is critical for establishing long-term programs and community-academic-government partnerships [19]. They also identified a need for additional training to support continuous and evolving COVID-19 education in minoritized communities, and noted time-limited funding reduced the efficacy of ramp-up/down efforts, long-term partnership and staff sustainability.

A hallmark of the GOTVax program was the flexibility for tailored community outreach that allowed CBO leaders to creatively provide culturally-, linguistically-, and socioeconomically-congruent outreach by canvassers themselves, residents of the communities, to reach the community for COVID-19 vaccine registration. For example, some CBOs in rural areas partnered with the California Department of Public Health to obtain mobile vaccination vans that followed canvassers to facilitate registration after vaccination, or others found local events (e.g., swap meets), through crowd-canvassing an optimal place to reach low-income, multi-lingual, high-risk populations. The canvassing model used in this program enabled CBOs to address other social needs affected by the pandemic, including rental relief and low-cost internet. This model promises to improve tailored community outreach by extending CBO services to address evolving community-specific needs.

### Public Health Implications

Lessons learned from CBOs participating in this program can inform approaches to future public health outreach and can be applied to other public health emergencies. First, the GOTVax CBO leaders identified a need for more efficient distribution of funds to CBOs and capacity-building to develop an infrastructure to manage funds. Second, the role of canvassers in community outreach holds promise for reaching high-risk communities in health endemics (e.g., diabetes and obesity), disaster response, and addressing social needs [31, 32]; however, this approach must incorporate strategies to address the physical, interpersonal, and technologic challenges associated with door-to-door outreach. Third, iterative feedback from the CBOs was essential to tailoring the GOTVax program design to meet the needs of local communities such as crowd-canvassing. Fourth, this program can serve as a blueprint for rapidly establishing effective community-academic-state partnerships.

This study has some limitations. First, the sample included CBO leaders in selected regions in one state, and the study findings may not be generalizable to CBOs in other regions of California or in other states. Second, the interviews occurred while COVID-19 vaccine eligibility, information, and access rapidly changed, which may have affected CBO leaders’ beliefs, attitudes, and concerns about their participation in the GOTVax program. Additionally, we did not include the perspectives of the individual canvassers, whose insights are important for understanding these processes. Nonetheless, this study provides insight into the barriers and facilitators to program participation perceived by CBO leaders.

## Conclusion

As government and academic institutions partner with CBOs in community-engaged outreach, it is vital to incorporate their perspectives into the design, implementation and management of outreach activities and modify outreach strategies to best support the CBO’s needs. To address future public health emergencies, public health programs should leverage partnerships with community-trusted CBOs, support CBO capacity building, and provide flexible funding for tailored outreach in local communities.

## Electronic supplementary material

Below is the link to the electronic supplementary material.

Supplementary Material 1

## Data Availability

All data generated or analyzed during this study are included in this published article and its supplementary information files.
