# Supplementary material for: Community-based organization perspectives on participating in state-wide community canvassing program aimed to reduce COVID-19 vaccine disparities in California
Source: BMC Public Health. 2023 Jul 14;23:1356. doi: 10.1186/s12889-023-16210-9 (PMC10349443; doi:10.1186/s12889-023-16210-9)
Supplement: Supplementary file 1 — Supplementary Material 1 [file 12889_2023_16210_MOESM1_ESM.docx]

**Appendix A**

**GOTVax Program Description**

The Get Out the Vaccine (GOTVax) program was a novel partnership between local community-based organizations (CBOs), the state of California, and the University of California, Los Angeles (UCLA), to reduce Coronavirus 2019 (COVID-19) vaccine disparities in vulnerable communities across the state. The primary goal of this program was to vaccinate eligible individuals by reducing structural barriers to COVID-19 vaccine registration and facilitating vaccination through on-the-ground community outreach in areas with hard-to-reach/underserved populations, communities of color, those with high occupational risk, e.g., communities with low vaccination rates despite high risk of COVID-19 infection, morbidity, hospitalization, and mortality.

Communities in high-risk zip codes were selected by the State of California that were: (a) disproportionately impacted by COVID-19, (b) in the lowest quartile of the California Healthy Places Index (HPI), a database that depicts community conditions that predict life expectancy and influence health (e.g., housing, education, environmental, and socioeconomic factors) [20] and (c) unreached by other previous State-led initiatives. Current vaccination rates were also assessed in the selection of communities participating in GOTVax. The State of California partnered with UCLA to select and approve CBOs to participate in the program based on whether they were located and served communities in the selected high-risk zip codes and had not received previous funding from other state COVID-19 outreach mechanisms. CBOs either had a previous relationship with the research team, and/or had previous canvassing experience through the Census, political campaigns, or voter registration.

The GOTVax program modeled a similar approach to Get Out the Vote (GOTVote) campaign, a political outreach approach that deploys community-based canvassing through door-to-door knocking, phone banking, and text messaging to register voters and increase voter turnout in elections. Using the GOTVote infrastructure, the GOTVax program mobilized 34 local CBOs who had deep knowledge of their communities, and longstanding trusting relationships with residents to perform grassroots canvassing through knocking door-to-door, crowd-canvassing in frequented locations (e.g., supermarkets, food banks), and phone banking. Using the GOTVote infrastructure also enabled UCLA to use the Political Data Intelligence (PDI) technology, mapping, engagement, and coordination software that gathered demographic data from registered voter files, census data, and commercial data and tracked the number of contacts made during canvassing (e.g., number of doors knocked, phone calls made, outcome etc.).

**Funding and Contracts**

The State of California Office of Government Operations and UCLA created a new and innovative funding mechanism that disbursed funds based on each CBOs’ performance and milestones achieved during the program. Funding through an academic institution typically follows a reimbursement model, which requires individuals to use personal funding and later receive reimbursed funds after providing documentation of approved purchases. Selected CBOs initially received full funding ($299,700) or half ($149,850) based on each CBO’s scope to facilitate canvassing strategies for COVID-19 outreach, education, and scheduling vaccine appointments, employ grassroots and community mobilization tactics, provide culturally and linguistically appropriate resources, and provide strategic project work plans and progress reports.

The GOTVax program was also an economic stimulation program where local CBOs hired unemployed and underemployed community canvassers. Hired canvassers reflected the people in the community or were residents themselves from communities being canvassed to registered local residents for vaccination, provide information about COVID-19 and vaccines, and enhance access to vaccination sites. CBOs were contracted to hire and deploy 25 part-time canvassers and two full-time CBO managers. Canvassers were paid roughly $22 an hour ($14 was CA minimum wage in Spring 2021), and GOTVax managers were paid over $25 an hour in high unemployment during the pandemic.

**Outreach Strategies and Materials**

Outreach activities, materials, and strategies were designed, developed, and implemented in collaboration with the CBOs, who were attentive to the linguistic, medical, and social needs of their communities. Outreach materials included COVID-19 vaccine frequently asked questions (FAQs) sheets in multiple languages, including Spanish, Mandarin, Cambodian, Armenian, Hmong, Lao, Vietnamese, and Korean. CBOs were provided in print and editable copies by UCLA for local CBOs to tailor materials, such as adding their logos and additional local information. CBOs also identified social needs and connected residents to appropriate resources. These strategies led to other outreach activities including coordinating transportation to vaccine sites, connecting residents to appropriate resources addressing social determinants of health (e.g., rental relief), and collaborating with other trusted local partners (e.g., churches, unions, other community-based organizations, and elected officials) to expand vaccine outreach.

**Academic and Government-Partnered Training Sessions**

Hired GOTVax canvassers and CBO managers participated in 4-hour online training sessions developed by UCLA investigators to educate and increase their knowledge about COVID-19 prevention and vaccination, effective messaging strategies using motivational interviewing to encourage individuals to vaccinate, how to respond to the community’s vaccine concerns, and updated COVID-19 information on prevention, vaccines, and variants. The training sessions were held in conjunction with Casitas Media and Strategic Group, a political consulting firm in Los Angeles that utilizes communications, messaging, and targeted allocation of resources for political campaigns, who provided training on canvassing tactics used in the GOTVax program and use of the PDI software. Training sessions were provided separately for GOTVax canvassers and CBO leaders in English or Spanish, and topics were refined and tailored to address CBOs’ and canvassers’ needs to address COVID-19 misinformation in their communities and reflect the evolving changes in local and national COVID-19 vaccine guidelines and social distancing and mask-wearing practices. A total of 13 training sessions were held (6 sessions for CBO leaders; 7 sessions for canvassers), where approximately 70-100 hired GOTVax staff attended each session. CBO leaders and GOTVax canvassers had ongoing access to the UCLA team for additional questions and updates about COVID-19 and vaccination.
